# Genital tract lesions in sexually mature Göttingen minipigs during the initial stages of experimental vaginal infection with *Chlamydia trachomatis* serovar D

**DOI:** 10.1186/s12917-016-0793-6

**Published:** 2016-09-10

**Authors:** Karin Erneholm, Emma Lorenzen, Sarah Bøje, Anja Weinreich Olsen, Peter Andersen, Joseph P. Cassidy, Frank Follmann, Henrik E. Jensen, Jørgen S. Agerholm

**Affiliations:** 1 Section of Veterinary Reproduction and Obstetrics, Department of Large Animal Sciences, Faculty of Health and Medical Sciences, University of Copenhagen, Frederiksberg C, Denmark; 2 Department of Infectious Disease Immunology, Statens Serum Institut, Copenhagen S, Denmark; 3 Present address: Timeline Bioresearch, Lund, Sweden; 4 Present address: Novo Nordisk A/S, Kalundborg, Denmark; 5 Pathobiology Section, School of Veterinary Medicine, University College Dublin, Belfield Dublin 4, Ireland; 6 Section of Experimental Animal Models, Department of Veterinary Disease Biology, Faculty of Health and Medical Sciences, University of Copenhagen, Frederiksberg C, Denmark

**Keywords:** Chlamydia, Sexually mature, Pig model, Porcine, Genital tract, Initial lesions

## Abstract

**Background:**

Chlamydia is one of the most common sexually transmitted diseases in humans worldwide, causing chronic lesions in the reproductive tract. Due to its often asymptomatic course, there is limited knowledge about the initial changes in the genital tract following infection. This study employs a novel sexually mature minipig model to investigate the initial histopathological changes following vaginal infection with *Chlamydia trachomatis* serovar D.

**Results:**

A vaginal inoculation resulted in an infection primarily affecting the lower genital tract. The histopathological changes were characterized by a subepithelial inflammation consisting of neutrophils and mononuclear cells, followed by an increase in the number of plasma cells within the sub-epithelial stroma of the vagina. Detection of *Chlamydia* was associated with expression of cyclooxygenase-2 and interleukin-8 by superficial epithelial cells. The infection was self-limiting, with a duration of 7 days.

**Conclusion:**

Neutrophils, plasma cells and IL-8 have been linked with *Chlamydia* genital infection of unknown duration in human patients. In this study, we observe a similar pattern of local immune response/inflammation following experimental inoculation suggesting this porcine model shows promise as a model for translational chlamydia research.

**Electronic supplementary material:**

The online version of this article (doi:10.1186/s12917-016-0793-6) contains supplementary material, which is available to authorized users.

## Background

Chlamydia is one of the most common sexually transmitted infections in humans, with more than 100 million new cases per year [1]. Genital infection with *Chlamydia trachomatis* (*C. trachomatis*) in women can lead to long term sequelae such as infertility, chronic pelvic pain and in worst case life threatening complications, e.g. ectopic pregnancy [2]. Our understanding of the pathogenesis of acute *C. trachomatis* infection in the genital tract of women is limited because the majority are asymptomatic [3]. Murine models are often used for translational studies of *Chlamydia* infections and in particular using the mouse pathogen, *Chlamydia muridarum* [4]. Models based on non-human primates (NHPs) are in general more comparative to humans than rodent models. Different studies, reviewed by Bell et al., have shown that a genital infection with *C. trachomatis* in NHPs elicits histological changes in the mucosa of the vagina, cervix and the oviducts, resembling the inflammatory response in humans [5]. However, pigs are increasingly being used as models for the study of human diseases as they are less expensive and more accessible laboratory animals than NHPs. Moreover, compared with rodents, porcine physiology and the porcine immune system are more comparable to humans [6–8]. Also, with respect to reproductive biology porcine models are preferred, as pigs are polyoestrous with a cycle length of 21 days; compared to a 5 day murine cycle [9]. Furthermore, the porcine vagina is suitable for both in vitro and in vivo studies due to the similarities between its structure and function with that in women [10, 11]. The use of pigs for experimental *Chlamydia* studies has been reported in a single study of a primary genital *C. trachomatis* infection, and in three vaccine studies [12–15]. In these studies, prepubertal gilts were used. No systematic evaluations of histological changes have been reported for early stages of infection in pigs or NHPs.

In humans, genital *Chlamydia* infection most often occurs in sexually mature adolescents [16]. The sexually mature genital tract differs from the immature tract, in size, epithelial thickness, vascularization, immune cell infiltration and hormonal fluctuation [17–20]. Given that hormones influence the susceptibility of endometrial cells to chlamydial infection [21, 22], sexually mature pigs should constitute a good model for the human disease.

In this study, we investigated the histopathological and immunological changes in the initial stages of infection with *C. trachomatis* serovar D (SvD) in sexually mature Göttingen minipigs, acting as a model of human genital chlamydia. The characterization included immunohistochemical staining of cyclooxygenase-2 (cox-2), a key enzyme for prostaglandin synthesis in the early inflammatory response in both pigs and humans, and interleukin-8 (IL-8), which has a similar function as proinflammatory cytokine and neutrophil chemoattractant in both pigs and humans, and is additionally known to be upregulated following *C. trachomatis* infection [23–26].

## Methods

### Chlamydia trachomatis

*C. trachomatis* SvD (Trachoma type D strain UW-3/Cx, ATCC® VR-885™) was propagated in HeLa cells as previously described [27]. Briefly, the HeLa cells were cultured in six well plates and infected with 1.5 *C. trachomatis* SvD inclusion forming units (IFU) per HeLa cell. The infection was followed by centrifugation at 750 g for 1 h, and thereafter incubated at 35 °C for 2 h, after which the media was enriched with 0.5 % glucose and 1 μg/ml cyclohexamide. The plates were incubated at 37 °C for 48 h, and thereafter harvested and purified as described by Olsen et al. [27].

The concentration of infectious bacteria was determined by culturing bacterial suspensions on McCoy-cells [28]. The plates were incubated at 37 °C for 22 h. Visualization of inclusions was made by incubating the cells with polyclonal rabbit antibodies against chlamydial Major Outer Membrane Protein and chlamydial Heat Shock Protein-60 [29], and thereafter stained with 4 μg/ml Alexa Fluor 488 labelled goat-anti rabbit antibody (Life Technologies) and kept in the dark at 4 °C until microscopy was carried out.

The cell plates were evaluated using a fluorescent microscope (Olympus IX71). Inclusion bodies were enumerated by counting positively stained inclusions in at least 20 fields in each well using the 40x objective on microscope. Results were calculated as average of duplicate samples.

### Experimental animals

Ten 7 to 8 month old sexually mature female Göttingen minipigs were raised in a barrier facility at Ellegaard Göttingen Minipigs A/S, Dalmose, Denmark, declared free of *Chlamydia* infection and a number of other infections as documented by a FELASA-approved health monitoring report. The experimental study was performed in the Laboratory Animal Isolation Unit at the University of Copenhagen. The gilts were housed in groups of 3–4 with wooden shavings as bedding material, fed twice daily with standard minipig diet and had ad libitum access to water. In addition, genital tracts were also obtained as histologic control tissue from six age-matched gilts, raised at the same facility as the experimental animals.

### Study design

**Fig. 1:**
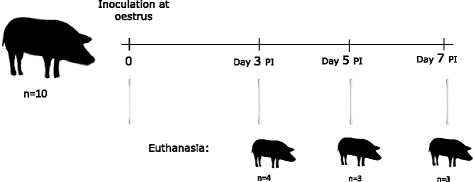
Experimental design. Ten sexually mature minipigs were intravaginally inoculated with *C. trachomatis* at oestrus. The pigs were euthanised and sampled on day 3, 5 and 7 post inoculation (PI). As controls for the histological stainings, genital organs from six age-matched, healthy, non-treated gilts were also included (not shown in the figure)

The study design is illustrated in Fig. 1. The gilts were treated orally for 18 days (20 mg/day) with a progestagen (Regumate Equine®, MSD Animal Health, Ballerup, Denmark) to synchronize their oestrus cycles and then evaluated daily for signs of oestrus. When characteristic signs of oestrus were evident, such as vulvar erythema, oedema, and increased physical activity (4–5 days after discontinuation of treatment), the pigs were anaesthetized with Zoletil-50® mixture [30] intramuscularly. Chlamydial inoculation was performed under sterile conditions using an insemination catheter (Osiris, E-vet, Denmark) placed in the most cranial portion of the vagina (external uterine orifice). The bacterial suspension deposited consisted of 1.8 × 10^9^ IFU in 5 ml of 250 mmol/l sucrose, 10 mmol/l NaH_2_PO_4_, and 5 mmol/l L-glutamic acid (SPG) buffer. The caudal part of each pig was slightly elevated for 20 min post-inoculation to minimize reflux of the inoculum.

Vaginal swabs were taken immediately prior to inoculation and at euthanasia. Moreover, vaginal swabs were collected on day 3 and 5 post inoculation (PI). The pigs were sedated during the sampling and sterile procedures were applied. The vaginal swabs were immediately placed in 1 ml SPG buffer and kept on ice until further processing. Glass beads were added to the sample tubes, and the samples were whirlmixed for 1 min. Fresh samples of 100 μl were used for culturing. The remainder of each sample was aliquoted in tubes and stored at -80 °C.

The pigs were clinically monitored daily. Any changes in behaviour, food intake, or in defecation/urination patterns were noted. Moreover, the vulva was inspected for erythema, oedema, and discharge. Rectal temperature was measured daily from the day before inoculation until euthanasia. Fever was defined as a rectal temperature >39.5 °C. On the day of inoculation, the rectal temperature was taken 6–9 h PI. The pigs were euthanized on days 3 (*n* = 4), 5 (*n* = 3), and 7 (*n* = 3) PI by deep anaesthesia followed by exsanguination.

### Necropsy

The pigs were examined for grossly visible lesions [31] and the entire reproductive tract was isolated and mucosal swabs from vagina, cervix, uterine corpus, uterine horns, and oviducts were taken and transferred into 1 ml SPG. Mucosal swabs were handled similarly to the vaginal swabs, were aliquoted and stored at -80 °C. Samples of vagina, cervix, uterine horns, and oviducts were fixed in 10 % neutral buffered formalin for 48 h prior to transfer into 70 % ethanol until further processing.

### Tissue processing and histological examination

Tissue samples were trimmed, processed routinely, and embedded in paraffin. Sections of 3–4 μm were cut, mounted on SuperFrost®/Plus glass (Hounisen, Denmark), deparaffinised and rehydrated by standard methods. For histopathological evaluation, the slides were stained with haematoxylin and eosin, and examined without prior knowledge of experimental treatment. The phase of the oestrous cycle was determined as “peri-oestrus” (ranging from pro-oestrus to early di-oestrus) or “dioestrus”, based on histological changes in the uterine horns; Peri-oestrus if the epithelium was pseudostratified, subbasilary bands of neutrophils were found, and apoptotic cells were detected in the endometrial and/or glandular epithelium, and dioestrus if no apoptotic cells and subbasilar neutrophils were found, the epithelium was columnar and the glandular layer hypertrophic [32, 33].

### Immunohistochemistry (IHC)

Selected sections of tissues of infected animals and control tissues were cut, deparaffinised and rehydrated by standard methods. To detect chlamydial antigen, all sampled areas of the vagina and cervix were examined; sections from the upper genital tract were also examined by IHC when inflammation was observed histologically and/or if the corresponding sample was positive by q-PCR. All sections of vagina and cervix were stained for IgM, IgA, IgG, cox-2, and IL-8. Antibody dilutions, antigen retrieval, quenching of endogenous peroxidase, blocking steps, and application of antibodies and enhancers were performed as detailed in Table 1 and according to the manufacturer’s protocol. The slides were counterstained with haematoxylin and mounted with an aqueous medium. TBS was used as washing buffer between the steps. Negative control-sections were included by replacing the primary antibody with a nonsense isotype-matched antibody from the same species. Positive control sections known to contain chlamydial antigen were included.

**Table 1:** Details of immunohistochemical protocols

Target antigen	Manufacturer	Dilution and incubation	Detection method and chromogen
Mouse anti-Chlamydia^a^	Progen Clone Acl-C	1:100 o/n RT	Vectastain Elite-ABC - Mouse
Mouse anti-pig CD3e^b^	Southern Biotech SB 4510-01	1:1000 o/n 4 °C	Ultravision One DAB
Goat anti-pig IgM^c^	Nordic Biosite Lot no: A100-100A-11	1:5000 1 h RT	Vectastain Elite-ABC – Goat AEC
Goat anti-pig IgA^c^	Nordic Biosite Lot no: A100-102A-16	1:4000 1 h RT	Vectastain Elite-ABC – Goat AEC
Goat anti-pig IgG-Fc^c^	Nordic Biosite Lot no: A100-104A-12	1: 7000 1 h RT	Vectastain Elite-ABC – Goat AEC
Rabbit anti-mouse/rat Cox-2^d^	Cayman chemicals Lot. no: 0428356-1	1:400 o/n 4 °C	Ultravision One AEC
Mouse anti-sheep IL-8^e^	Abd Serotec Clone 8 M6, MCA 1660	1:1000 o/n 4 °C	Ultravision LP AEC

For antigen retrieval, the following procedures were used: boiling in ^a^Citrate buffer pH 6.0 for 2 × 5 min ^b^TEG buffer pH 9.0 for 2 × 5 min, ^c^Citrate buffer pH 6.0 for 5 min ^d^TED buffer pH 9.0 2 × 5 min. For ^e^IL-8, a DIVA decloaker solution in a pressure cooker (2100 retriever, Biocare Medical) was used. Blocking for endogenous peroxidase was carried out using a solution of hydrogen peroxide (0.6 %) for 15 min for all stainings except Chlamydia, where a 1 % dilution for 30 min was used

IgM, IgA and IgG-positively stained cells in the vaginal and cervical mucosa were counted in a zone 100 μm from the basal layer; at least 20 fields were counted with a 20x objective. The average number of antibody expressing cells/mm^2^ was calculated. Similar staining and counting was performed in sections from the corresponding anatomical locations in the uninfected controls. Cox-2 and IL-8 expressing epithelial cells were counted in the whole section, and the length of the epithelium measured using A Cell software (Olympus). Cellular expression was measured as numbers of positive cells/mm epithelium. Similar staining and counting was performed in the control specimens.

### Vaginal chlamydial load by culturing

Culturing of vaginal swabs was made by a titrated inoculation of McCoy cells [28], with some minor adaptions, equivalent to the previously described procedure for determining bacterial concentrations.

### DNA extraction and q-PCR detection of *C. trachomatis*

DNA extraction from the swabs was performed with Chelex®100 (Bio-Rad, Life Science, Denmark). Swab material (100 μl) was mixed with 300 μl of a 20 % Chelex solution in TE buffer (T9285, Sigma Aldrich), vortexed for 60 s and incubated at 96 °C for 10 min. The sample was then centrifuged for 10 min at 17,500 g at 4 °C, diluted 1:10 and hereafter triplicates of 5 μl of the supernatant were used for q-PCR.

Real-time q-PCR detection of *C. trachomatis* in the vaginal swab samples was performed by detection of the 16S rRNA gene. An internal control (IC) was systematically run to detect possible inhibition of the PCR. The following primers and probes were used: *C. trachomatis* 16 s-F GGATCTTCGGACCTTTCGGT; *C. trachomatis* 16 s-R AATCTCTCAATCCGCCTAGACA; *C. trachomatis* 16 s-probe FAM-AAGGGAGAGTCTATGTGATAT – MGBNFQ (Applied Biosystems); IC-F 5′ACCGCTCAGGCATTTGCT-3′; IC-R 5′CCGGGACGTATCATGCT3′ (TAG Copenhagen A/S, Copenhagen, Denmark), IC-probe NED-TCCTTCGTGATATCGGACGTTGGCTG- MGBNFQ (Applied Biosystems). The assay was performed with a final reaction volume of 20 μl with: Perfecta q-PCR SuperMix (UNG, low ROX, 95066-02 K (2000 rx) Quantum Biosciences), 300 nM of each primer, 75 nM of each of the probes, IC-DNA and DEPC treated water was added up to a total volume of 20 ul. The samples were run on a StepOne™ Real-time PCR instrument (Applied Biosystems®) programmed to run 2 min at 95 °C and 40 cycles of denaturation at 95 °C for 15 s and annealing/ extension at 60 °C for 1 min. The C_t_ cutoff was determined to be 38 hence C_t_ values greater than 38 were considered negative.

### *Chlamydia*-specific antibody response in serum

An indirect enzyme-linked immunosorbent assay (ELISA) was used to evaluate the *C. trachomatis* specific antibodies in serum. Polysorp® plates (NUNC A/S, Roskilde, Denmark) were coated with UV-inactivated *C. trachomatis* SvD elementary bodies (EBs) (4 μg/ml) over night at 4 °C. For blocking and dilution of secondary antibodies a PBS buffer with 1 % casein and 0.05 % Tween 20 was used. *Chlamydia* specific IgM, IgG and IgA were detected with HRP-conjugated antibodies; goat-anti-pig IgM (AAI39P, Serotec, UK in 1:10000), goat-anti-pig IgG∙Fc (AAI41P, Serotec, UK in 1:10000) and goat-anti-pig IgA (AAI40P, Serotec, UK in 1:2000). The reactions were visualized with TMB PLUS substrate (Kem-En-Tec, Taastrup, Denmark) and stopped with 0.5 M sulphuric acid. The plates were read on an ELISA reader at 450 nm with correction at 650 nm. Confirmed positive serum from an earlier study was included as a positive control and two wells were run without substrate as a negative control on each plate. Each sample was run in duplicates in serial dilutions. The positive control was used as an internal standard to correct for plate-to-plate variation. The corrected OD values are presented as the increase in OD-value on day x PI (3, 5 and 7 PI) compared to day 0 PI (∆OD_day(x)PI_ = OD_day(x)PI_ -OD_day0PI_). The dilution was 1:640 for IgM analyses and 1:10 for IgA and IgG analyses.

### Statistical analyses

Statistical analyses were performed using GraphPad Prism 5. Differences in mean rectal temperatures were analysed with a Repeated measures ANOVA, using Tukey’s multiple comparison post test. Kruskal Wallis test was used with Dunn’s multiple comparison post test to compare plasma cell infiltrations. Spearman’s correlation was used for determining correlation between cox-2 and IL-8. A cut off value for elevated expression was used; corresponding to the mean value of inoculated animals +3 standard deviations, after a test for outliers was performed. A value of *P* < 0.05 was considered statistically significant.

## Results

### Clinical signs and necropsy findings

All pigs remained clinically unaffected during the study period. As expected, all pigs developed signs of oestrus, which decreased over time, and on day 4 PI, vulvar erythema and oedema had resolved. The mean rectal temperature increased from 38.5 °C to 39.1 °C 6–9 h PI. On day 1 PI, the mean rectal temperature had returned to a normal level. The temperature variation was statistically significant (Additional file 1). All genital tracts exhibited oedematous endometria and slight to moderate hyperaemia of the broad ligament. One pig (day 3 PI) had a thin-walled cyst, 7 × 7 × 7 mm, with clear fluid, adjacent to the infundibulum in the mesosalpinx, considered to be a mesonephric duct remnant.

### Histopathology

The oestral cycle stage was found to be peri-oestral in eight out of ten experimental animals (80 %), and in five out of six of the genital control organs (83 %) based on the histological appearance, while two pigs from day 7 PI, and one genital control organ were in dioestrus.

**Fig. 2:**
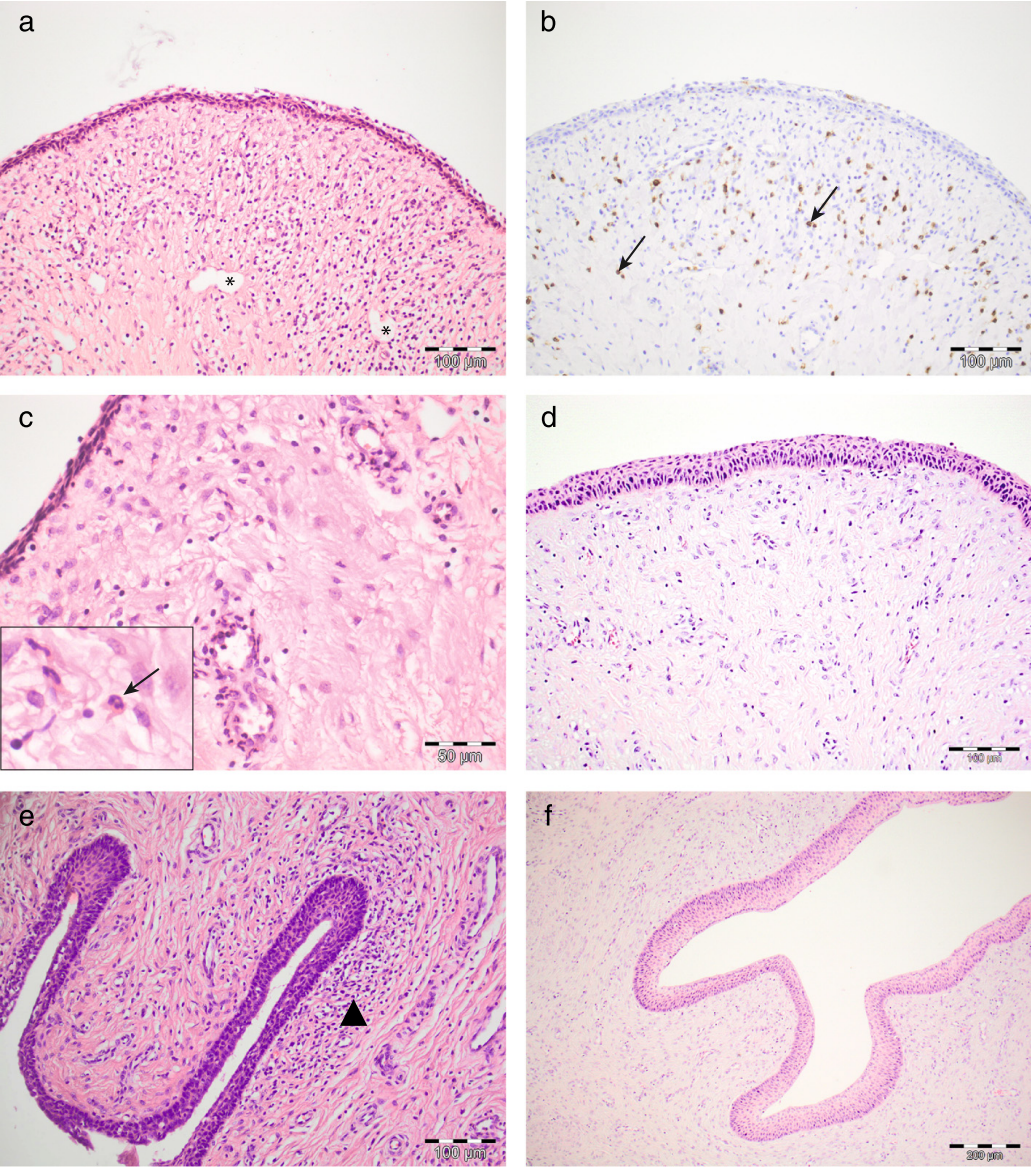
Histopathological changes in the lower genital tract. **a** Vagina, day 3 PI, haematoxylin and eosin (HE), showing subepithelial stromal and perivascular infiltrations of mononuclear, lymphoid cells, and dilated lymphatic vessels (*); **b** Vagina, day 3 PI (serial section of a): IHC staining of CD3 showing positive staining in some, but not all of the infiltrating cells (*arrows*); **c** Vagina, day 3 PI, HE, showing neutrophils extravasating in the subepithelial stroma (enlarged in insert, *arrow*); **d** Vagina, HE, control tissue; **e** Cervix, day 5 PI, HE, showing a dense subepithelial infiltrate of mononuclear cells (*arrowhead*); **f** Cervix, HE, control tissue

**Fig. 3:**
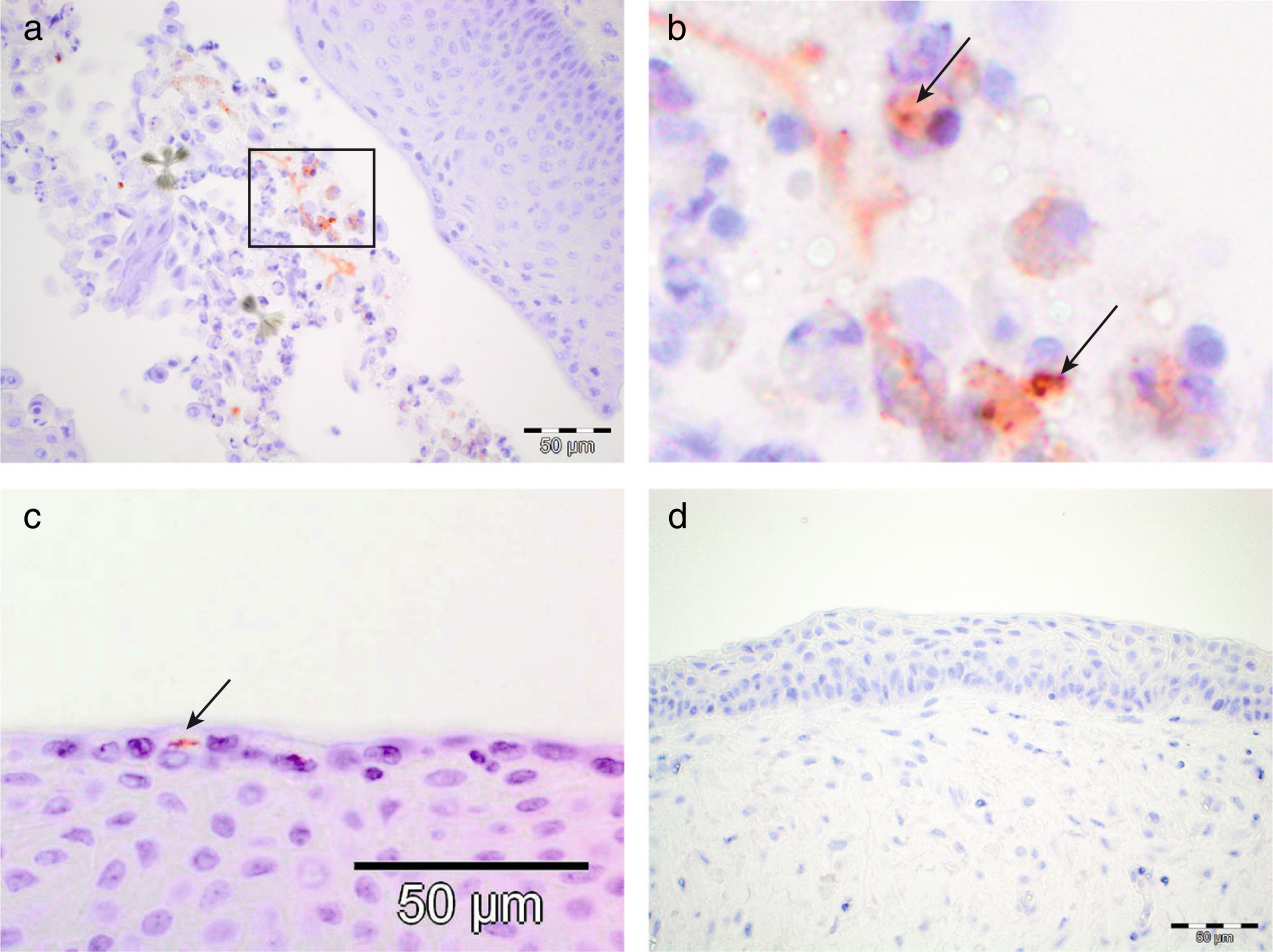
IHC staining of *Chlamydia*. **a** Vagina, day 3 PI. Positive intracellular staining within a luminal accumulation of sloughed epithelial cells, neutrophils and macrophages; **b** Higher magnification of the high-lighted area in **a**) showing positively stained material intracellularly located; **c** Vagina, day 3 PI, showing an inclusion in a superficial epithelial cell (*arrow*); **d** Vagina from control tissue

On day 3 PI, an increased number of mononuclear, lymphoid cells, of which some were positive for CD3, were present in the subepithelial stroma of the vagina and cervix of experimentally infected pigs (Fig. 2a, b). Perivascular infiltrates of lymphocytes, occasionally in large numbers were noted especially in the vagina. Perivascular infiltrates were not noted in any of the control tissues (Fig. 2d, f). Some of the mononuclear cells showed features of hydropic degeneration. Neutrophils were found in the epithelium and in the subepithelial stroma, predominantly perivascularly (Fig. 2c). Stromal oedema and dilated lymphatics were also noted (Fig. 2a). Mild to moderate amounts of cell debris, including sloughed epithelial cells, neutrophils and macrophages were present in the vaginal and cervical lumen. Slight amounts of luminal contents, primarily consisting of epithelial cells, were also found in the vagina and cervix of a single control pig. Chlamydial antigen was detected by IHC in epithelial cells and intracellularly in luminal debris in the vagina of two of the four pigs (Fig. 3 a, b, c), and in the cervix of one pig. Uterine changes were sparse, and consisted of small stromal clusters of mononuclear cells, and mild stromal infiltration of neutrophils. On day 5 PI, mild infiltrations of mononuclear cells were present subepithelially in the vagina and cervix (Fig. 2e), while perivascular infiltrations were found only in a single pig. A low number of mononuclear cells were noted in the uterine stroma. Chlamydial antigen was detected by IHC in two of three vaginal samples. On day 7 PI, there was a slight increase in diffuse lymphoid cell infiltrations in the vagina and cervix. Mild perivascular infiltrations were noted, and plasma cells were found in the subepithelial stroma. Chlamydial antigen was not detected in any of the control tissues by IHC (Fig. 3d).

**Fig. 4:**
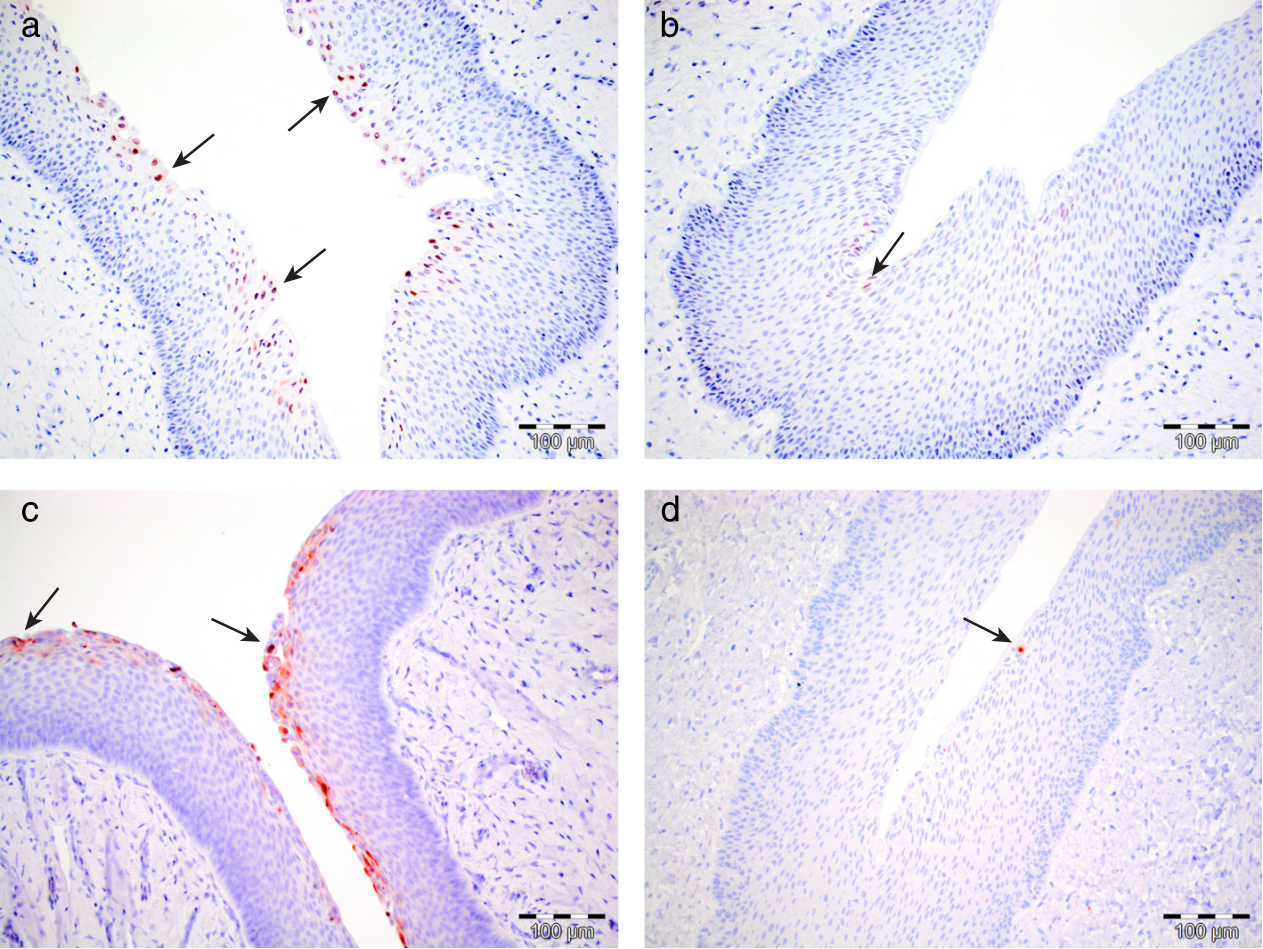
IHC staining of cox-2 and IL-8 in vaginal tissue from day 3 PI, and control tissues. **a** Vagina, day 3 PI. Positive staining in numerous superficial epithelial cells for cox-2 (*arrows*); **b** Vagina from control tissue, showing positive staining for cox-2 in single superficial epithelial cells (*arrow*); **c** Vagina, day 3 PI. Positive (*red*) staining in numerous superficial epithelial cells for IL-8 (*arrows*); **d** Vagina from control tissue showing positive staining for IL-8 in a single epithelial cell (*arrow*)

**Fig. 5:**
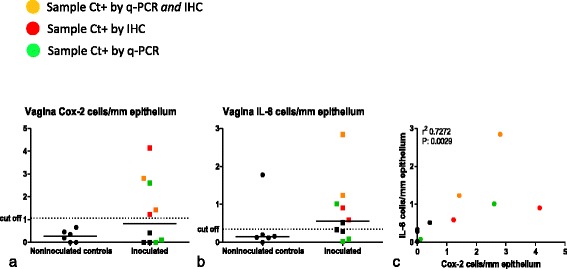
Expression of cox-2 and IL-8 by epithelial cells in the vagina. Numbers of cox-2 (**a**) and IL-8 (**b**) expressing cells/mm epithelium of the uninoculated histological control tissues, compared to inoculated experimental animals. Cut off: mean of controls +3SD. **c** Correlation of cox-2 and IL-8 expression by vaginal epithelial cells (Spearman). The colours mark the samples testing positive for *Chlamydia* by either q-PCR and IHC (*orange*), q-PCR alone (*green*) or IHC alone (*red*)

Inoculated pigs showed elevated numbers of cox-2 and IL-8 positive cells in the vaginal epithelium (Figs. 4a-d and 5a, b). Expression of cox-2 and IL-8 was significantly correlated (Fig. 5c). In addition, samples with elevated levels of cox-2/IL-8 corresponded to samples positive for *Chlamydia* by IHC and/or q-PCR (Fig. 5a-c). Three of ten cervical samples had elevated levels of cox-2, and two samples exhibited elevated levels of IL-8.

### Detection of *C. trachomatis* by culture and q-PCR

**Fig. 6:**
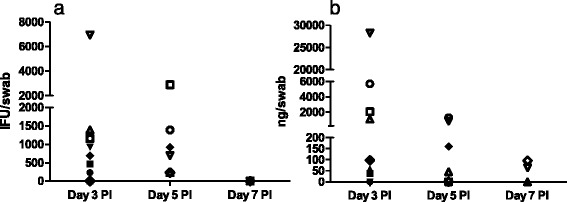
Detection of *C. trachomatis* in vaginal swabs. **a** cell culturing (shown as IFU/swab); **b** q-PCR (shown as ng of *C. trachomatis*/swab). Results are shown for single pigs

Culturing of vaginal swabs revealed replicating *C. trachomatis* on day 3 and 5 PI (Fig. 6a). Similarly, q-PCR identified *C. trachomatis* DNA in vaginal swabs on the same days (Fig. 6b). Both culture and q-PCR showed the highest amounts of *Chlamydia* on day 3 PI (Fig. 6a-b). Only a few samples were positive on day 7 PI. *C. trachomatis* DNA was detected by q-PCR in uterine samples on all three sampling days, however with a variation in distribution and amount (Table 2). Similar to the vaginal samples, most antigen was found on day 3 PI, with decreasing amounts detected on days 5 and 7 PI. Positive samples were also detected in the oviducts in individual pigs (Table 2).

**Table 2:** Detection of *C. trachomatis* by q-PCR in different parts of the genital tract at days 3, 5 and 7 PI

	Day 3 PI				Day 5 PI			Day 7 PI		
Pig number	1	2	3	4	5	6	7	8	9	10
Vagina	0	+	+	0	++	+++	0	0	+	+
Cervix	+	+	0	+	0	0	0	0	+	+
Uterine body	+++	0	+	0	0	0	0	0	0	0
Left uterine horn, lower part	+	0	+	0	+	0	0	0	0	+
Left uterine horns, higher part	+	0	+	0	+	+	0	0	0	+
Left oviduct	0	0	+	0	+	0	0	0	0	+
Right uterine horn, lower part	++	0	+	0	0	0	0	0	0	0
Right uterine horn, higher part	++	0	+	+	0	0	0	0	+	0
Right oviduct	+	0	0	0	0	0	0	0	0	+

0: Below detection limit (C_t_ >38), +: 1–100 ng/swab, ++: 101–1000 ng/swab, +++: >1000 ng/swab

### Antibody-producing cells in the vagina and cervix

**Fig. 7:**
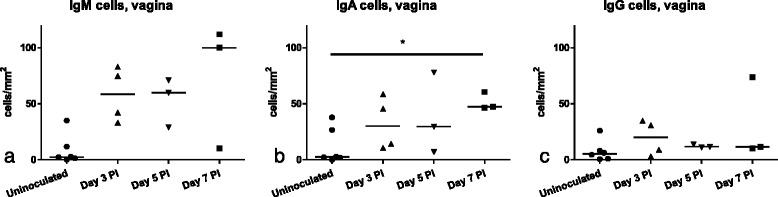
Numbers of IgM, IgA and IgG-expressing cells in the vagina, compared to control tissues. Positively stained cells were counted to a stromal depth of 100 μm from the basal epithelial layer, and measured as numbers of cells/mm^2^. **a** IgM **b** IgA and **c** IgG. Lines show medians. Statistics: Kruskal Wallis, Dunn’s multiple comparison post test

A tendency for increased numbers of IgM and IgA expressing cells was found in the vagina on all days PI compared to control tissues; however, the difference was only significant regarding IgA^+^ cells on day 7 PI (Fig. 7a-c). In the cervix, there were no differences in the number of antibody positive cells in the infected pigs compared to controls. A small amount of *Chlamydia*-specific antibodies of all three isotypes was detected in serum, predominantly of IgM isotype (Additional file 2).

## Discussion

A novel model of human genital *Chlamydia* infection was evaluated in sexually mature Göttingen minipigs. Infection and the related tissue changes were primarily found in the lower genital tract, which is also the most common site of human chlamydial infections [34, 35]. In humans, the duration of a primary genital *C. trachomatis* infection is difficult to assess because up to 80 % of infected women are asymptomatic [36]. A follow-up study in women has shown spontaneous clearance in 18 % of detected cases, in 13 days from the time of sampling until treatment [37]. Although a time-dependent correlation of clearance was found, lack of knowledge remains about the host response following uncomplicated genital infection with *C. trachomatis* [38]. In addition, no systematic evaluations of the initial histopathologic changes have been reported from large animal studies of *C. trachomatis* pathogenesis.

In our study, the most striking histological changes were subepithelial clusters of numerous lymphoid cells in the vagina on day 3 PI. It is known from murine in vivo and human in vitro studies that natural killer (NK) cells are quickly activated and involved in IFN-y clearance of *Chlamydia* infections [39], thus a proportion of the infiltrating cells are thought to be of NK cell origin, further supported by the heterogenous expression of CD3 by the infiltrating lymphocytes (Fig. 2b). The CD3-positive cell population could consist of either NK-T cells [40], or unspecific CD4/CD8 T cells attracted to the site of inflammation. IHC staining of cox-2 and IL-8 showed positive staining in superficial vaginal and cervical epithelial cells in locations where cells positive for *Chlamydia* on IHC were also found (Fig. 5a-c). This corresponds to what is described in humans as IL-8 is secreted by *Chlamydia*-infected human cervical epithelial cells in vitro [23]*,* and has further been shown to be up regulated in the cervicovaginal fluid of *Chlamydia*-infected women [41]. Similarly, cox-2 is an enzyme related to pro-inflammatory responses that can be induced in human cervico-vaginal cells as a response to toll-like receptor (TLR)-ligands and TNF-α, as demonstrated in an in vitro model of vaginal infection [26].

Neutrophils were found intraepithelially and within the subepithelial stroma, reflecting an ongoing innate immune response. Surprisingly, there were no neutrophils in immediate association to the chlamydial inclusions in the epithelium, as would have been expected with the observed increase in IL-8 expression, a well recognised neutrophil chemoattractant. However, positive *Chlamydia* staining was found intracellularly in luminally located clusters of epithelial cells, neutrophils and macrophages, indicating an innate immune reaction to the infection (Fig. 3a, b). Infiltration with neutrophils was one of the typical histological changes described in the intestine of *Chlamydia*-infected gnotobiotic piglets four days PI [42]. However, this feature was not consistently observed in association with chlamydial antigen, as described in the current study. In women, intraepithelial and intraluminal neutrophils have also been associated with various endometrial pathogens, including *C. trachomatis* [43, 44] indicating a similar host response in our *C. trachomatis* minipig model.

By detecting expression of IL-8 and cox-2 by epithelial cells, associated with chlamydial antigen, and with attendant infiltration of neutrophils and probable NK cells, we have demonstrated pro-inflammatory and innate immune responses in this novel experimental model. It is likely that these responses are responsible for the decreased shedding of bacteria observed within a week of inoculation. The innate immune response has previously been shown to clear *C. trachomatis* infection without involvement of the adaptive immune system in a murine model [45].

In the current study, a rapid decrease in the number of replicating bacteria in the vagina occurred within one week of inoculation. In addition, bacteria were detected by q-PCR in endometrial and oviduct samples, on all sampling days, although it was not possible to find inclusions by IHC in the more cranial regions of the genital tract. This could be due to a combination of a rapidly decreasing bacterial load and the sampling of an insufficient number of locations. This finding did not correlate with other porcine studies, in which replicating bacteria were detected in the genital tract of prepubertal pigs for up to 25 days following inoculation [13]. The observed differences may be explained by the different *C. trachomatis* serovars used, and also the sexual maturity of the pigs. It is known that sex hormones influence the innate and adaptive immune response of the female reproductive tract [20, 46], and the susceptibility of its epithelial cells to *C. trachomatis* [21, 47]. As chlamydial infections in women are most common in sexually mature women, the sexually mature Göttingen minipig constitutes a more comparable model of the human disease than models using sexually immature animals. Further optimisation, to establish a long term infection, are currently taking place in our laboratory. Future studies could also include repeated inoculations, as reinfection is one of the risk factors thought to be associated with an adverse outcome in *Chlamydia* infected women [2].

In the vagina, a tendency towards larger numbers of IgM^+^ and IgA^+^ cells were found, however the difference was only significant for IgA^+^ cells on day 7 PI (Fig. 7b). We cannot determine the antigenic specificity of these cells, but it is likely that they were recruited as a response to infection. Plasma cell infiltrates are one of the typical hallmarks of a chlamydial infection in women [48], and our results indicate a similar pattern in *Chlamydia*-infected pigs. We only detected a slight antigen specific antibody response in serum (Additional file 2), probably due to the early sampling points in relation to an initiation of a systemic adaptive response. Other studies by our group, using the same basic study design, have revealed a *Chlamydia*-specific adaptive immune response at day 12 PI [49], and an adaptive immune response following a genital *Chlamydia* infection has also been demonstrated using prepubertal pigs [14].

In this study, we included control specimens of age-matched, sexually mature gilts for the histological evaluation. These gilts were not subjected to the same procedures as the experimental gilts, including hormonally synchronisation, vaginal inoculation and swab sampling procedures. Therefore differences in handling may have caused discrepancies. However, as the majority of the genital tract organs from both the experimental and control groups were in peri-oestral stage, we consider the hormonal influences on the mucosa to be comparable between the groups. There is a risk of inducing changes to the mucosa following inoculation and sampling procedures due to mechanical disruption and reaction to the inoculum buffer solution. However, in earlier studies in mice sham-inoculated with SPG, no histological changes were detected in the genital tract (Erneholm, unpublished observation). Therefore, we do not think that SPG itself and/or the inoculation and sampling procedures would give rise to considerable changes in the genital tract of the pigs. In addition, the findings of increased levels of cox-2 and IL-8 in the experimental group were co-localised to areas with detection of chlamydial antigen, which further supports our hypothesis that this reaction is a consequence of the *C. trachomatis* infection.

## Conclusion

A promising sexually mature minipig model of human genital *C. trachomatis* infection has been developed that generates comparable initial immune responses to those reported in human patients. The porcine model has several advantages to other animal models, as it is more comparable to humans than mice, and is cheaper and more accessible compared to NHP. Further studies using this model have the potential to generate further insights into the early pathogenesis of *C. trachomatis* infection.

## Additional files

Additional file 1: Body temperatures (^o^C). Data show mean rectal temperatures and whiskers represent 95 % confidence intervals. At day 0, the mean temperature was significantly increased compared to all other time points. (PDF 47 kb) Additional file 2: Serum antibodies. Detection of serum antibodies against* C. trachomatis*, measured as the difference in OD values day (x) and day 0. (PDF 57 kb)
