# Supplementary figures and images for: Genital tract lesions in sexually mature Göttingen minipigs during the initial stages of experimental vaginal infection with Chlamydia trachomatis serovar D
Source: BMC Vet Res. 2016 Sep 10;12(1):200. doi: 10.1186/s12917-016-0793-6 (PMC5018167; doi:10.1186/s12917-016-0793-6)

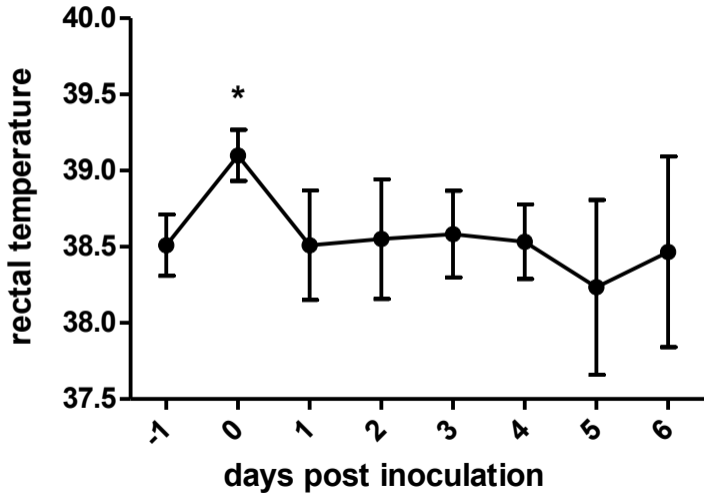

Supplement: Additional file 1: — Body temperatures (oC). Data show mean rectal temperatures and whiskers represent 95 % confidence intervals. At day 0, the mean temperature was significantly increased compared to all other time points. (PDF 47 kb) [file 12917_2016_793_MOESM1_ESM.pdf]

IgM

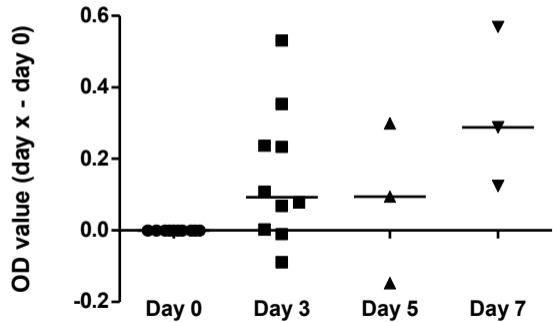

IgA

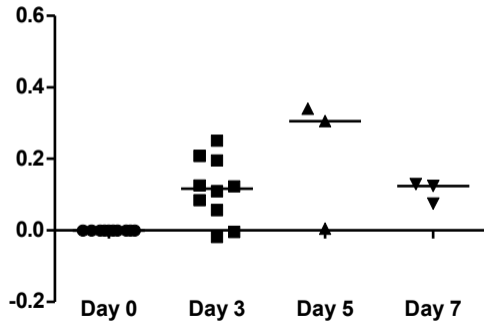

IgG

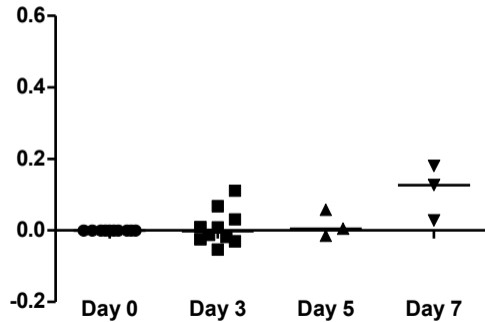

Supplement: Additional file 2: — Serum antibodies. Detection of serum antibodies against C. trachomatis, measured as the difference in OD values day (x) and day 0. (PDF 57 kb) [file 12917_2016_793_MOESM2_ESM.pdf]
